# Antiphase Synchronization in a Flagellar-Dominance Mutant of *Chlamydomonas*

**DOI:** 10.1103/PhysRevLett.111.158101

**Published:** 2013-10-08

**Authors:** Kyriacos C. Leptos, Kirsty Y. Wan, Marco Polin, Idan Tuval, Adriana I. Pesci, Raymond E. Goldstein

**Affiliations:** 1 Department of Applied Mathematics and Theoretical Physics, University of Cambridge, Wilberforce Road, Cambridge CB3 0WA, United Kingdom; 2 Mediterranean Institute for Advanced Studies (CSIC-UIB), E-07190 Esporles, Spain

## Abstract

Groups of beating flagella or cilia often synchronize so that neighboring filaments have identical frequencies and phases. A prime example is provided by the unicellular biflagellate *Chlamydomonas reinhardtii*, which typically displays synchronous in-phase beating in a low-Reynolds number version of breaststroke swimming. We report the discovery that *ptx1*, a flagellar-dominance mutant of *C. reinhardtii*, can exhibit synchronization in precise antiphase, as in the freestyle swimming stroke. High-speed imaging shows that *ptx1* flagella switch stochastically between in-phase and antiphase states, and that the latter has a distinct waveform and significantly higher frequency, both of which are strikingly similar to those found during phase slips that stochastically interrupt in-phase beating of the wild-type. Possible mechanisms underlying these observations are discussed.

Living creatures capable of motion seldom restrict themselves to a single mode of propulsion. Pairs of appendages of multilegged organisms can be actuated synchronously in phase, out of phase, or asynchronously by a “central pattern generator” [1]. In the world of aquatic microorganisms, where there is no central nervous system, the cilia and flagella adorning algae and bacteria are the “limbs” which exhibit various sychronization modes, generating swimming [2]. Within a given eukaryotic organism, the motor-driven undulations of flagella can be found to synchronize in two stereotypical ways. Biflagellates epitomized by the alga *Chlamydomonas* [3] display synchronous beating with identical frequencies and phases [4,5]. Those with multitudes of cilia or flagella, such as unicellular *Paramecium* [6] or multicellular *Volvox* [7], exhibit metachronal waves in which flagellar phases vary monotonically with position. Theory [8–10] suggests that these modes of synchronization can arise from fluid dynamical coupling between flagella, possibly assisted by waveform compliance.

Flagellar synchronization is more complex than the simplest models of coupled oscillators would suggest; beating is intrinsically stochastic, cells can switch between synchrony and asynchrony [5], and flagella within a single organism can be functionally distinct. These features are well established for *Chlamydomonas*; the flagella of wild-type (*wt*) cells typically exhibit a noisy in-phase (IP) breaststroke [Fig. 1(a)]. Termed *cis* and *trans* for their proximity to the cell’s eyespot, the two flagella are differentially affected by calcium, exhibiting a tunable flagellar dominance [11] important in phototaxis.

We report here an alternative mode of synchronization found in eukaryotes, in which flagella lock in antiphase (AP) synchronization. For a range of conditions [12], this behavior can be sustained in time by the “flagellar-dominance” mutant *ptx1* of *Chlamydomonas reinhardtii* [13]. This mode of synchronization in *ptx1* was described qualitatively by Rüffer and Nultsch as “parallel coordination” [14] in contrast to “bilateral coordination” (IP), referring to the relative motion of the flagellar bases as determined from light table tracings of frames of short (~ 4 s) high-speed movies, with minimal quantitative analysis. While *ptx1* cells exhibit no gross motility defects, they have defective phototaxis [13–15] thought to arise from a lack of Ca^2+^-dependent flagellar dominance. We discuss mechanisms proposed for AP synchronization [8,16–19], and suggest that our observations support active filament models [20] which exhibit discrete undulating modes of flagella.

Wild-type (CC125) and *ptx1* (CC2894) strains [21] were grown photoautotrophically in Tris-minimal medium [22] with revised trace elements [23] and air bubbling in a diurnal growth chamber at 24 ° C on a 14:10 h light-dark cycle with a light intensity of 90 *μ*E m^−2^ s^−1^ [5]. Cells were harvested from 1- or 2-day-old cultures at a density ~6 × 10^5^ cells/ml, during hours 4 and 5 of the day, washed with buffer HKC-40/10 [24], and allowed to regrow flagella for at least 2 h. Cylindrical PDMS chambers (15 mm ⊘ ×4 mm height), cast in aluminum molds and plasma etched onto 22 × 50 mm cover slips, were placed on a Nikon TE2000-U microscope with a ×63 Plan-Apo water-immersion objective (Carl Zeiss AG, Germany). Cells were held and oriented by micropipettes [5]. Bright-field illumination utilized a halogen lamp with a long-pass filter (> 620 nm) to minimize phototactic behavior during experiments, which were performed without background illumination. Video microscopy was performed at 1000 fps (Fastcam SA3, Photron, U.S.), and postprocessed in MATLAB. After each recording the filter was removed to locate the orange-colored eyespot and identify the *cis* and *trans* flagella. Experiments with *wt* cells showed that *Chlamydomonas* need to be acclimated for ≳ 20–30 min before characteristic synchronized breaststrokes are observed [4,5]. Data from 10 *wt* cells and 12 *ptx1* cells were analyzed.

There are four key observations. First is the existence of the AP state itself [Fig. 1(d)], visualized by discrete wave-forms within one cycle, color coded in time, and overlaid on a spatial map of average flagellar residence time. Compare this to Fig. 1(a) showing the *wt* IP breaststroke. Here, the flagella simultaneously execute extended “power strokes” followed by high-curvature “recovery strokes,” in which they are drawn forward with distal portions sliding past the body. In the AP of *ptx1*, distinct power and recovery strokes are clearly discernible, but as one flagellum executes the former, the other proceeds through the latter. The mutant also displays an IP state [Fig. 1(c)] that is nearly [12] identical to the *wt* IP. For example, the areas $A_{\text{IP}}^{wt,ptx1}$ swept out by the flagellum in both cases (i.e., the areas within residence-time plots in Fig. 1) agree to within 1%. In the case of *ptx1*, evident also is the drastic reduction in spatial extent spanned by both flagella during AP relative to the *wt* IP mode. This alteration of beating waveform occurs with an abrupt increase in beating frequency, which together comprise our second observation. We extract flagellar phases *ψ*_cis,trans_ from Poincaré sectioning of the dynamics [5] and define the interflagellar phase difference as Δ = (*ψ*_trans_ − *ψ*_cis_)/2*π*. For a typical *ptx1* cell, Fig. 2(a) tracks Δ(*t*) over ~40 s as it fluctuates around half-integer values during AP, but around integer values during IP. As seen in Fig. 2, our third finding is that flagella of *ptx1* stochastically transition between IP and AP modes, in a manner reminiscent of the synchronous or asynchronous transitions of the *wt* [5]. Figure 2(b) shows that the instantaneous beat frequency is indeed higher in AP (*ν*_AP_: 82 ± 4 Hz) than in IP (*ν*_IP_: 58 ± 5 Hz). Fourth, we highlight the striking similarities between the AP state and that of the flagellum that accumulates one additional cycle during a phase slip of the *wt* [5], as evidenced by the equivalence of the waveforms [Figs. 1(b) and 1(d), areas $A_{\text{slip}\;}^{wt},\;A_{\text{AP}}^{ptx1}$ agree to within 5%], and the frequencies [Figs. 2(c) and 2(d)]. The latter figure shows also the similarity of *wt* and *ptx1* IP beat frequencies.

The hypothesis that there is a second, distinct beating mode can be explored through estimates of the flagellar force *F* and power *P* [25]. In a caricature of the power stroke we imagine a straight flagellum of length *L* pivoting from initial polar angle *θ*_0_ to a final one *θ*_*f*_ during half the beat period. Using resistive force theory we integrate the viscous force along the filament to obtain *F* ~ 2*ζ*_⊥_ *ν*𝒜, where *ζ*_⊥_ is the perpendicular drag coefficient and 𝒜 is the waveform area defined previously. A similar calculation yields the power *P* ~ (2/3)*FV*, where $V=L\dot{\theta }$ is the flagellum tip speed. Ratios of the product *ν* 𝒜 thus serve as measures of relative force in different beats. Restricting to a subset of cells whose flagella were most planar, averaged values of the pairs (*ν*, 𝒜) for the four states of interest are *ptx1* IP (57.2 Hz, 147.3 *μ*m^2^), *ptx1* AP (81.0 Hz, 105.1 *μ*m^2^), *wt* IP (59.4 Hz, 148.8 *μ*m^2^), *wt* slip (82.0 Hz, 110:1 *μ*m^2^). We find $F_{\text{IP}}^{ptx1}/F_{\text{AP}}^{ptx1}=0.99\pm 0.06$ and $F_{\text{IP}}^{wt}/F_{\text{slip}\;}^{wt}=0.98\pm 0.07$. The quantitative match of these ratios supports the identification of a *wt* slip with the transient appearance of a higher mode, and the fact that the common value is accurately unity implies equal force generation in the two states. Intriguingly, the ratio of the average AP and IP frequencies for *ptx1* and of the average slip and IP frequencies of the *wt* are nearly identical at ~4/3.

The polar angles (*θ*_cis_, *θ*_trans_) measured from the cell midline to equivalent points on the two flagella define a phase space with which to quantify synchrony. Figures 3(a) and 3(b) show IP and AP motion in this space for a single cell and a multicell average. Individual cells orbit fairly close to the diagonals, but the mean displays remarkably precise IP and AP motion, with phase coherence maintained during power and recovery strokes. Transitions to and from these two types of synchrony [Figs. 3(c) and 3(d)] are always initiated by one flagellum, either *cis* or *trans*, which undergoes alteration of beating mode first [12]. Using Poincaré sections we examine the reemergence of synchrony during transitions between the modes using the difference (*ψ*_lead_ − *ψ*_follow_)/2*π* between the phase of the flagellum that leads the transition and that which follows. On a phenomenological level AP → IP and IP → AP transitions should obey a noisy Adler equation [5]:

(1) $$\dot{\Delta }=-V^{\prime }(\text{Δ})+\xi (t).$$

Here, *V*(Δ) = − *δν*Δ + *U*(Δ), with *δν* an intrinsic frequency difference and *U* an effective potential periodic in Δ, and *ξ*(*t*) is a noise term. Applying this to either type of synchrony in *ptx1* we expect *δν* ≃ 0 due to the lack of flagellar dominance [15]. The most parsimonious model would then be *U* = − ***ϵ*** cos(2*π*Δ), with ***ϵ*** > 0 for AP → IP and ***ϵ*** < 0 for IP → AP. Solving for the deterministic dynamics (*ξ* = 0) in a scaled time $s=\bar{\nu }\left(t-t_{i}\right)$ centered at the inflection point of the transition *t*_*i*_, where $\bar{\nu }$ is the average IP frequency, we obtain Δ = − (1/2*π*)cos^−1^ tanh(*s*/*τ*), with rescaled relaxation time *τ* = 1/(4*π*^2^***ϵ***/*ν*). Fits to the data yield *τ*_AP→IP_ = 1.65 ± 0:02 and *τ*_IP→AP_ = −2.07 ± 0:04 [Figs. 3(c) and 3(d)] and thus $\epsilon_{\text{AP}\to \text{IP}}/\bar{\nu }≃0.015$ and $\epsilon_{\text{IP}\to \text{AP}}/\bar{\nu }≃-0.012$, consistent with the *wt* [5].

The necessity to invoke couplings of opposite sign to account for the AP and IP states within the simplest model (1) provides a natural starting point for a discussion of mechanisms proposed for synchronization. Two key issues arise: the structure of the potential *U* and the origin of the coupling constants. With *δν* = 0, the solution to the Fokker-Planck equation for the probability distribution function *P*(Δ) associated with (1) gives *βU* = − log[*P*(Δ)], with *β* related to the noise in the usual manner. The function *βU* so determined [26] will be a bistable potential with local minima at integers and half-integers. This could be accommodated by higher-order Fourier components, as *U*(Δ) ≃ − *ϵ* cos(2*π*Δ) − *α* cos(4*π*Δ), with *ϵ* > 0 and *α* > ***ϵ***/4. An alternative to this picture of a fixed potential landscape *U*(Δ) with stochastic hopping between local minima is a fluctuating landscape switching between potentials *U*_IP_ and *U*_AP_, the former with minima only at integers, the latter at half-integers. Within the limitations of a phase-oscillator description, the distinction between these views is fundamentally a matter of which degrees of freedom are considered part of the dynamical system and the relative time scales for those variables. In fact, precedent for a fluctuating landscape can even be seen in the *wt* [5], in which asynchronous beating (“drifts”) corresponds to a washboard potential tilted by a large *δν* so there are no local minima, while synchronous beating has *δν* small enough to allow local minima.

Models of synchronization based on hydrodynamic coupling often represent flagella by microspheres driven by an internal force. That force may be constant along a trajectory with elastic compliance [9], or the trajectories are rigid and the forcing varies with phase [8]. The mechanism of synchronization in the first class is illustrated in Figs. 4(a) and 4(b). Measuring the phases (*ϕ*_1_, *ϕ*_2_) as indicated, cilia are modeled as corotating orbits, say $\omega_{1}\equiv {\dot{\phi }}_{1}>0$ and $\omega_{2}\equiv {\dot{\phi }}_{2}>0$. If sphere 1 lags 2, then the flow produced by 1 will push 2 to a larger radius. If the internal force is constant, ${\dot{\phi }}_{2}$ will decrease, and 1 catches up. Conversely, if 1 leads 2, then it pushes 2 inward, so 2 acquires a higher phase velocity and catches up. The flow induced at 1 by 2 leads to consistent results, showing that corotating IP motion is stable. To model *Chlamydomonas* the spheres must be counterrotating, with say *ω*_1_ > 0 and *ω*_2_ < 0. Then, these considerations, together with anisotropy of the Stokeslets, predict stable AP synchronization. Indeed, the coupling constant in (1) scales as ***ϵ*** ^∝^ − *ω*_1_*ω*_2_ and is negative (positive) for co- (counter)rotation. In this simple model the AP beating of *ptx1* is the “normal” behavior and the IP mode is anomalous. The situation is not so clear, for if the relationship between radius and phase velocity is reversed, then the coupling changes sign [16,17]. This relationship could be influenced by mechanosensitive cues [27]. In the class of models with forcing that varies with phase angle, synchronization can be understood by similar means in terms of the flow induced by one sphere at the other. Allowing for noncircular trajectories as well as proximity to a no-slip surface leads to the possibility of an effective potential with the higher-harmonic structure discussed above, stabilizing both IP and AP patterns [8,19]. The difficulty in determining the relevance of these arguments to *ptx1* is that the two modes of synchronization are associated with distinct waveforms, with potentially different compliances, internal forcing, and proximity to the cell surface. A third model [18] builds on the fact that transient deviations from locked phases lead to yawing motion of the cell which can produce differential forces on the flagella, bringing them back into phase. While such a mechanism may pertain to free-swimming cells, it is not immediately clear how it can encompass the appearance of both IP and AP states of cells held firmly on micropipettes, where we observe only minute angular displacements (below 1 ° in both states). The presence of the cell body itself appears not to be essential for synchrony of the two flagella, for a *wt*-like breaststroke has been observed in isolated flagellar apparati (axonemes still connected through their basal bodies), after reactivation by ATP [28].

No existing models of eukaryotic flagella explain the antiphase waveform. Approaches based on optimizing swimming efficiency or nutrient uptake in a model of *Chlamydomonas* [29] do find a mode comparable to the IP state. Perhaps the AP waveform is not optimal in any conventional sense, but instead exists as one of a discrete number of modes that can emerge from sliding filament models [20]. It will be important to establish whether the higher frequency and distinct waveform are properties intrinsic to a single flagellum or derive from interactions between the two; key insight may be gained from examining dynamics of uniflagellated double mutants of *ptx1*.

The physiology of stochastic transitions in the pattern of flagellar beating is currently unknown; we hypothesize that fluctuations in the concentration of a small molecule or ion might be the origin. One candidate would be Ca^2+^, which in isolated and reactivated flagellar axonemes is known to control the waveform [30]. Interestingly, calcium ions are also responsible for the contractility of striated fibers that connect the basal bodies of flagella [31], which in turn may act as a spring with variable stiffness. The current state of this potential spring may influence the preferred mode of synchronization. Indeed, generalizing the orbiting-sphere model [9] to include an elastic connection between flagella bases can lead to stabilization of either IP or AP modes [Fig. 4(c)], depending on microscopic details. In the simplest linear spring, for example, the AP mode can be selected, for it is the mode in which the relative displacements of the flagellar connections within the cell body are most nearly constant. The role of these fibers for flagellar synchronization may be clarified by altering their mechanical properties by chemical or other means.

## Figures and Tables

**Fig. 1:**
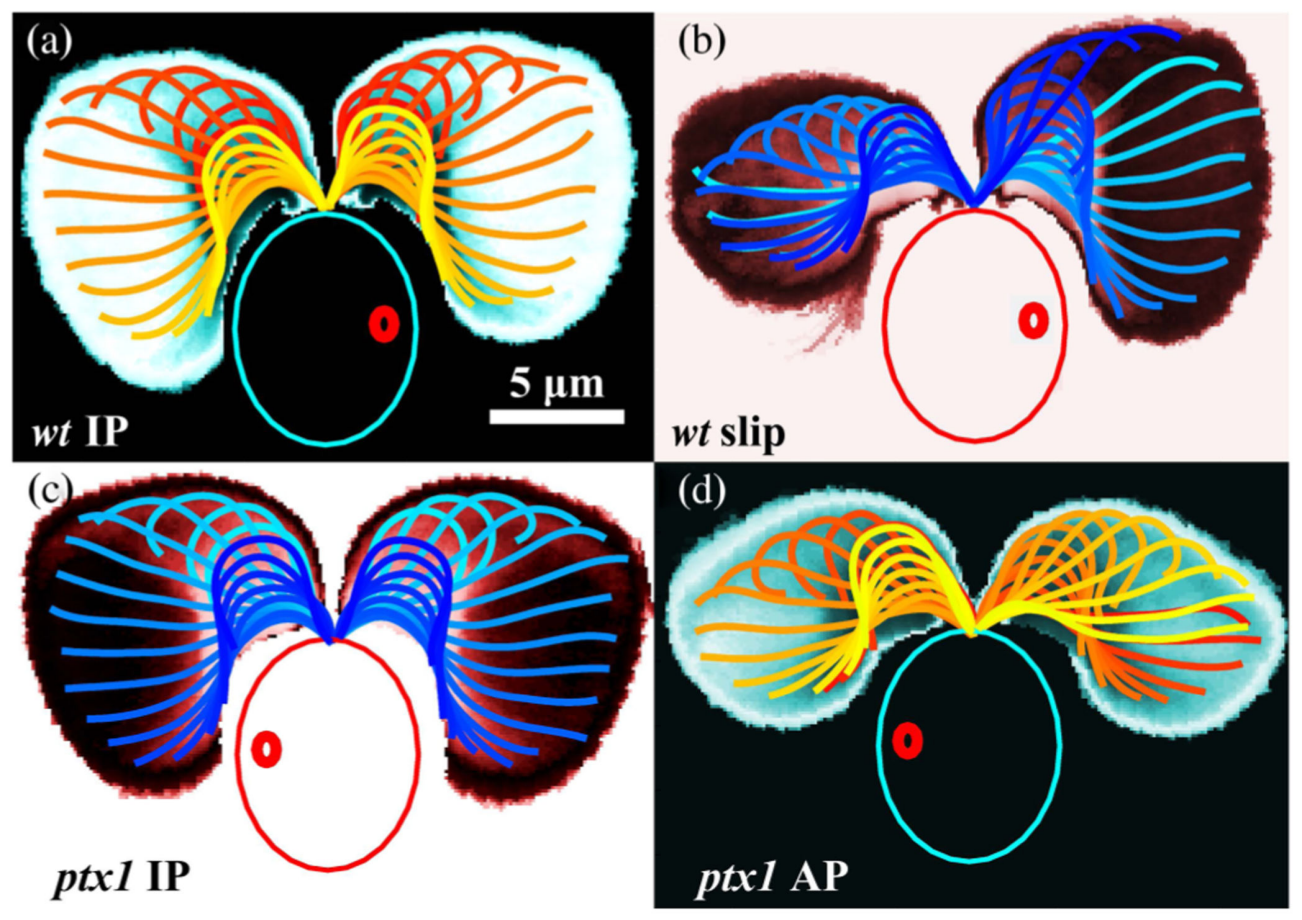
Waveforms of *C. reinhardtii*. Logarithmically scaled residence time plots averaged over 𝒪(10^2^) beats overlaid by waveforms, color coded in time. The *wt* displays IP breaststroke beating (a) stochastically interrupted by phase slips (b) in which one flagellum (here, *trans*) beats faster with an attenuated waveform. *ptx1* displays an IP state (c) nearly identical to the wild-type (a) and a high-frequency AP state (d). Large and small ovals indicate cell body and eyespot, respectively.

**Fig. 2:**
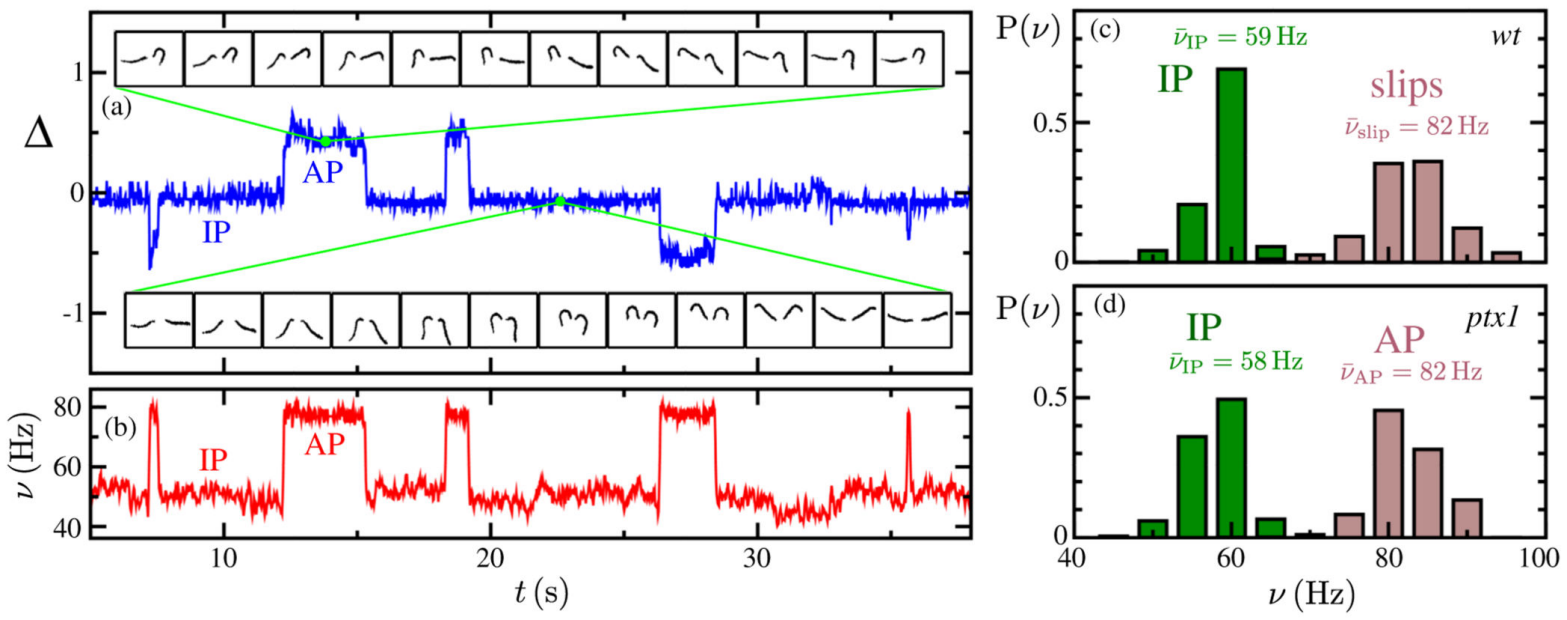
Beating dynamics. (a) Phase difference Δ = (*ψ*_trans_ − *ψ*_cis_)/2*π* showing half-integer jumps between IP and AP states. Insets show waveforms in the two states. (b) Instantaneous frequencies of AP and IP states. (c) Distribution of instantaneous frequencies during IP beating and of the faster flagellum during slips, across all sampled *wt* cells. (d) IP and AP instantaneous frequency distributions, across all sampled *ptx1* cells.

**Fig. 3:**
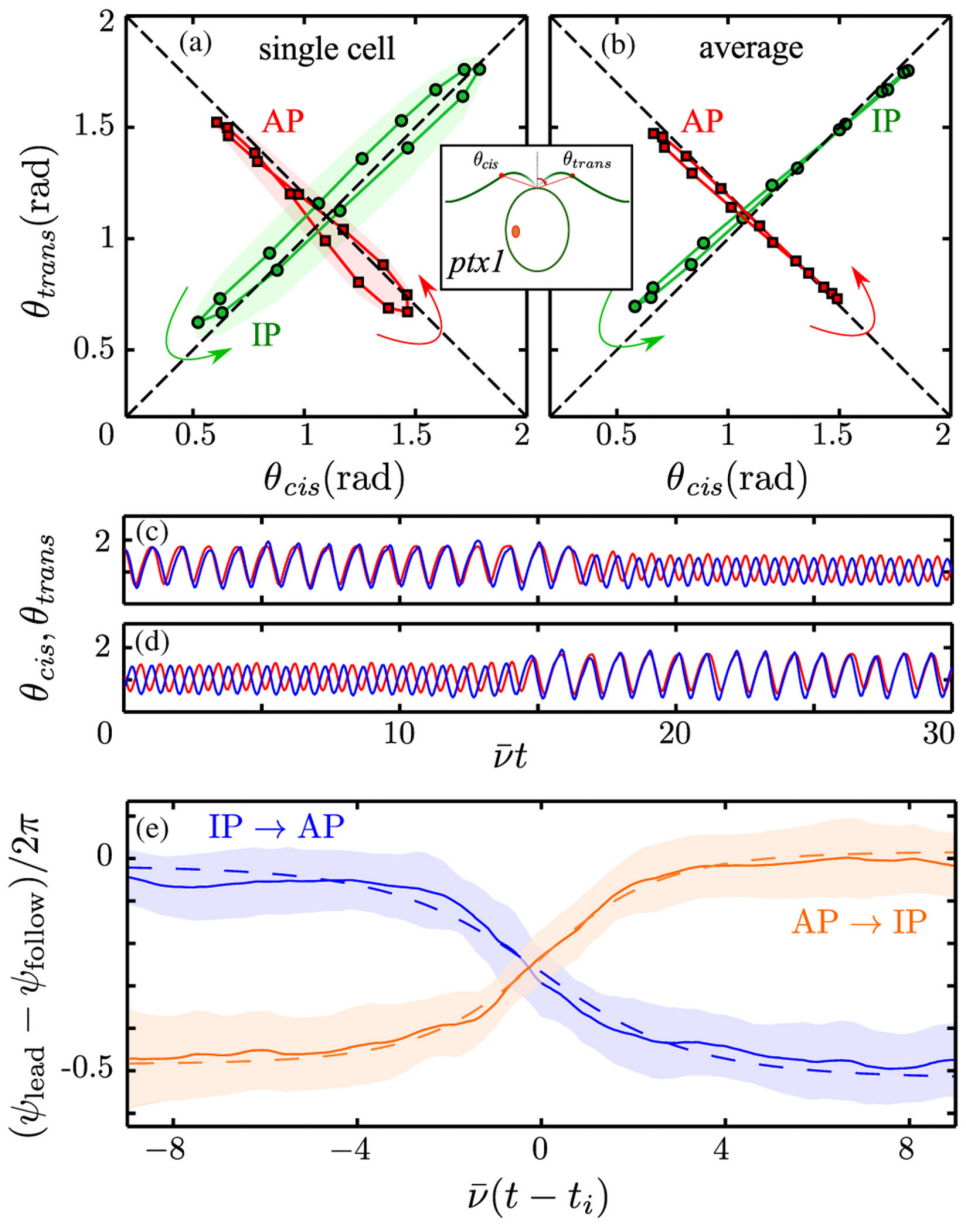
Synchronization dynamics. Phase plane of polar angles *θ*_cis,trans_ reveals the IP (green) and AP (red) synchronization of a single cell (a), and (b) the average over six cells, averaged over 𝒪(10^3^) beats and resampled at 15 points, equally spaced in time. Shaded regions in (a) indicate 1 standard deviation of fluctuations. (c),(d) Sample time series for evolution of *θ*_cis,trans_ during a transition event. (e) Phase difference dynamics during AP → IP (orange) and IP → AP (blue) transitions for 60 events, with means (solid lines) and standard deviations (shaded), vertically aligned by plotting difference modulo 1. Dashed lines are fits to data.

**Fig. 4:**
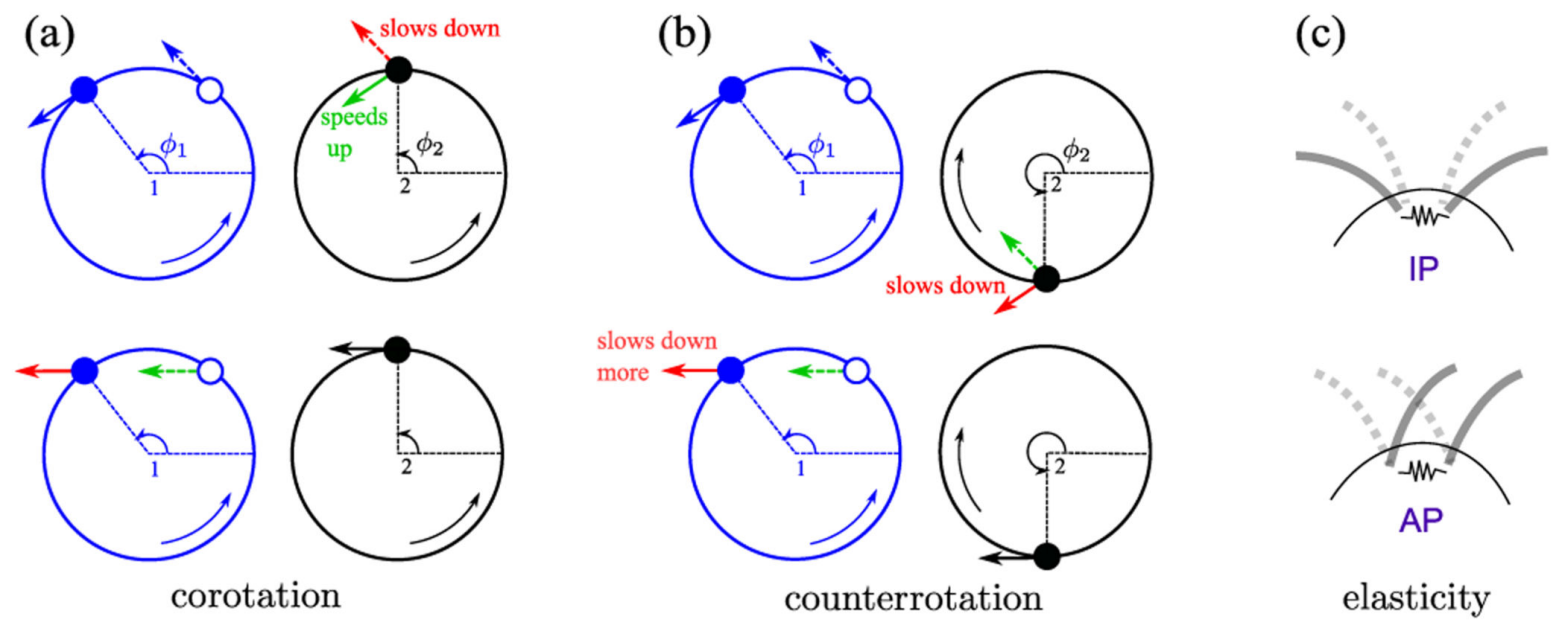
Synchronization mechanisms. (a,b) Top row: Motion of sphere 1 at two possible phases *ϕ*_1_ (solid and open circles) induces flows (blue arrows) which alter the trajectory of sphere 2, either speeding it up (green), or slowing it down (red). (a,b) Bottom row: Converse perspective. (c) Elastic coupling between flagella can induce either IP or AP modes.
